# Teneligliptin Co-Infusion Alleviates Morphine Tolerance by Inhibition of Spinal Microglial Cell Activation in Streptozotocin-Induced Diabetic Rats

**DOI:** 10.3390/antiox12071478

**Published:** 2023-07-24

**Authors:** Yaswanth Kuthati, Vaikar Navakanth Rao, Wei-Hsiu Huang, Prabhakar Busa, Chih-Shung Wong

**Affiliations:** 1 Department of Anesthesiology, Cathy General Hospital, Taipei 106, Taiwan; yaswanthk1987@gmail.com (Y.K.); cgh17178@cgh.org.tw (W.-H.H.); prabhakar.busa01@gmail.com (P.B.); 2 PhD Program in Pharmacology and Toxicology, School of Medicine, Tzu Chi University, Hualien 970, Taiwan; vaikarn@ibms.sinica.edu.tw; 3 National Defense Medical Center, Institute of Medical Sciences, Taipei 114, Taiwan

**Keywords:** diabetic neuropathic pain, dipeptidyl peptidase-4 inhibitor, teneligliptin, microglia, morphine antinociceptive tolerance

## Abstract

Morphine (MOR) is a commonly prescribed drug for the treatment of moderate to severe diabetic neuropathic pain (DNP). However, long-term MOR treatment is limited by morphine analgesic tolerance (MAT). The activation of microglial cells and the release of glia-derived proinflammatory cytokines are known to play an important role in the development of MAT. In this study, we aimed to investigate the effects of the dipeptidyl peptidase-4 inhibitor (DPP-4i) teneligliptin (TEN) on MOR-induced microglial cell activation and MAT in DNP rats. DNP was induced in four groups of male Wistar rats through a single intraperitoneal injection of streptozotocin (STZ) (50 mg/kg, freshly dissolved in 5 mmol/L citrate buffer, pH 4.5). Sham rats were administered with the vehicle. Seven days after STZ injection, all rats were implanted with an intrathecal (i.t) catheter connected to a mini-osmotic pump, divided into five groups, and infused with the following combinations: sham + saline (1 µL/h, i.t), DNP + saline (1 µL/h, i.t), DNP + MOR (15 µg/h, i.t), DNP + TEN (2 µg/h, i.t), and DNP + MOR (15 µg/h, i.t) + TEN (2 µg/h, i.t) for 7 days at a rate of 1 μL/h. The MAT was confirmed through the measurement of mechanical paw withdrawal threshold and tail-flick tests. The mRNA expression of neuroprotective proteins nuclear factor erythroid 2-related factor (Nrf2) and heme oxygenase-1 (HO-1) in the dorsal horn was evaluated by quantitative PCR (qPCR). Microglial cell activation and mononucleate cell infiltration in the spinal cord dorsal horn were assessed by immunofluorescence assay (IFA) and Western blotting (WB). The results showed that co-infusion of TEN with MOR significantly attenuated MAT in DNP rats through the restoration of neuroprotective proteins Nrf2 and HO-1 and suppression of microglial cell activation in the dorsal horn. Though TEN at a dose of 2 μg has mild antinociceptive effects, it is highly effective in limiting MAT.

## 1. Introduction

Neuropathic pain (NP) is one of the most common complications in both type 1 and type 2 diabetes mellitus (DM) and is estimated to affect more than 90% of patients [1]. DNP patients characteristically present symptoms of electric shock, numbness, tingling, burning, or stabbing types of painful sensations. The fundamental mechanisms of DNP are complex and comprise multiple mechanisms, such as oxidative/nitrative stress damage, chronic immune activation, impaired redox signaling, and downregulation of antioxidant mechanisms [2]. The currently available first-line treatment options for DNP include tricyclic antidepressants (TCAs), gabapentanoids (GBPs), serotonin norepinephrine reuptake inhibitors (SNRIs), transdermal drugs, and topical ointments. The second-line treatment options include drugs such as tapentadol and tramadol. Opioids/serotonin-specific reuptake inhibitors (SSRIs)/anticonvulsants and *N*-methyl-D-aspartate (NMDA) antagonists are often used as a last line of treatment option in patients with moderate to severe DNP [2]. Though opioids such as MOR are very effective in the treatment of DNP, long-term exposure leads to analgesic tolerance [3,4].

Prolonged MOR exposure leads to the activation of microglial cells through different immune signaling pathways [5]. The activation of microglial cells results in the release of proinflammatory cytokines (PICs) such as interleukin (IL)-1β, tumor necrosis factor (TNF)-α, and IL-6 [6]. PICs can hyperactivate the neurons in the dorsal horn and induce reactive oxygen species (ROS) generation leading to central sensitization and MOR tolerance [7]. Considering the key role of spinal microglial cell activation in MOR tolerance, it can be hypothesized that the attenuation of glial cell activation can attenuate MOR tolerance. This approach can efficiently interrupt the signaling pathways which ultimately cause MOR tolerance. Consistent with this, several microglial cell inhibitors such as metformin, minocycline, resveratrol, and curcumin have been shown to be effective in attenuation of MOR tolerance [8,9,10,11]. Chronic MOR exposure not only upregulates oxidative stress pathways but can also downregulate the efficacy of natural antioxidative enzyme mechanisms within the body [12,13].

Several reports have confirmed that the co-administration of exogenous antioxidants along with MOR can alleviate analgesic tolerance. Antidepressants like venlafaxine and fluoxetine with antioxidant properties attenuated MOR tolerance [14,15]. Natural antioxidants such as curcumin and bergamot were also proven to be effective in the alleviation of MOR tolerance [11,16,17]. Thus, the rational design of combinational drug therapy delivers the opportunity to overcome opioid tolerance.

Existing literature suggests that dipeptidyl peptidase-4 inhibitors (DPP-4is) have multiple pleiotropic properties that can protect multiple organs including the kidney, brain, liver, and heart from ROS damage [18,19,20,21]. TEN has distinct pharmacokinetic properties with a distinct chemical structure from other DPP-4is [22,23]. TEN was recently approved for the management of type 2 diabetes mellitus [24]. Apart from the inhibition of DPP-4, TEN displayed moderate analgesic effects against hyperalgesia in humans [25]. Additionally, TEN is known to possess important pleiotropic properties that are independent of DPP-4 inhibition. For instance, TEN is shown to improve endothelial dysfunction by enhancing endothelium-derived nitric oxide synthase (NOS) in vivo [26]. TEN is proven to be effective in the prevention of atherosclerosis through the suppression of monocyte chemoattractant protein-1 expression along with enhanced antioxidant enzyme production within the cells [27]. TEN is also known to possess more ·OH scavenging ability than naturally occurring antioxidant glutathione through direct ·OH scavenging [28]. The antioxidant properties of TEN are DPP-4-independent, as TEN showed antioxidant properties in DPP-4-deficient animals [28]. Remarkably, other DPP-is in the same category did not exhibit free radical scavenging abilities [28]. Several studies have demonstrated that TEN has remarkable anti-inflammatory effects through the suppression of PICs [29,30,31,32]. Furthermore, DPP-4 is known to be significantly overexpressed by microglial cells during NP [33]. Intrathecal application of DPP4-is like tripeptide isoleucin-prolin-isoleucin and vildagliptin showed a remarkable anti-hyperalgesic effect in the animal models of NP [33]. In our previous study, we demonstrated that TEN alleviates partial sciatic nerve transection-induced NP through the suppression of spinal astroglial cells [34].

These investigations indicate the effectiveness of TEN in the amelioration of neuropathological conditions in which microglial cells and PICs play a crucial role. Considering the role of microglial cells and PICs in the development of MOR tolerance along with the inhibitory effects of DPP-4is on glia cell activation and PICs, we hypothesized that teneligliptin might attenuate MOR tolerance in DNP.

In this study, we explored the efficacy of TEN in the attenuation of MOR tolerance and investigated the involvement of microglial cells and proinflammatory cytokines in the spinal cord.

## 2. Materials and Methods

### 2.1. Reagents

TEN was purchased from MCE (Princeton, NJ, USA). MOR, STZ, and dimethyl sulfoxide (DMSO) were acquired from Sigma-Aldrich (St. Louis, MO, USA). TEN and MOR were prepared in 4% DMSO. DMSO did not show any significant analgesic effects or toxic effects on animals at the concentrations used in our study [35,36]. An Accu-check instant glucometer was purchased from Roche Diagnostics (Mannheim, Germany). The anti-CD11B antibody was purchased from Abcam [EPR1344] (ab133357) (Cambridge, UK).

### 2.2. Animals

The procedures used in our animal experiments were approved by the ethical committee for animals at the National Defense Medical Center, Taipei, Taiwan, and are in accordance with the standards set by the National Institute of Health Guide for the Care and Use of Laboratory Animals (IACUC-19-222). Male Wistar rats were obtained from BioLASCO, Taipei., Taiwan.

### 2.3. Induction of Diabetes Mellitus

DM was induced by injecting a single intraperitoneal injection of newly prepared STZ in 0.01 M citrate buffer adjusted to pH 4.5 at a dosage of 60 mg/kg body weight. After overnight fasting, blood was collected from the rat tail veins through a puncture with a syringe needle. Rats with blood glucose higher than 250 mg/dL were considered as diabetic.

### 2.4. Intrathecal Catheterization and Osmotic Pump Infusion

Intrathecal catheters were implanted under 2% isoflurane inhalation. The cisternal material was carefully sliced to insert a polyethylene catheter (PE 10 tubes, 8.0 cm) into the spinal cord. The open end was connected with an osmotic pump (Alzet 2001, Cupertino, CA, USA) in five groups and infused with the following combinations: sham + saline (1 µL/h, i.t), DNP + saline (1 µL/h, i.t), DNP + MOR (15 µg/h, i.t), DNP + TEN (2 µg/h, i.t), and DNP + MOR (15 µg/h, i.t) + TEN (2 µg/h, i.t) for 7 days at a rate of 1 μL/h. After osmotic pump installation, rats were kept in individual cages and provided with a 12 h light/dark cycle along with food and water. Rats with neurological problems were sacrificed immediately.

### 2.5. Behavior Test for Tactile Allodynia

The paw withdrawal sensitivity was evaluated in the left posterior paw with a Dynamic Plantar Aesthesiometer (Ugo Basile, Comerio, Italy). Rats were enclosed in plastic chambers mesh beneath. After 30 min of acclimatization, the withdrawal threshold was measured with a progressive increase in weight in the range of 1 to 50 g through a blunt metal rod (0.5 mm) facing the plantar region of the paw. The procedure was repeated thrice with 2 min intervals and averaged by setting 50 g as a cut-off threshold.

### 2.6. Spinal Cord Sample Preparation for Western Blotting Analysis

On day 7 after osmotic pump installation, animals were sacrificed using isoflurane anesthesia (Abbott Laboratories Ltd., Queenborough, Kent, UK), and the spinal cords were stored at −80 °C until further use. The samples were lysed by ultrasonication (Misonix, Inc. Farmingdale, NY, USA) in a lysis buffer, followed by centrifugation at 15,000 RPM for 30 min at 5 °C. The supernatant was carefully isolated and quantified. Proteins were denatured by heating at 90 °C for ten minutes and isolated by 12% SDS-PAGE. Further, they were transmitted onto a polyvinylidene fluoride membrane (PVDF) (Pall, Ann Arbor, MI, USA) and blocked with 5% skimmed milk in Tris-buffered saline (0.05% Tween 20 in Tris-buffered saline). The PVDF membrane was incubated overnight with primary antibody at 4 °C: anti-GAPDH (1:2000; Santa Cruz Biotechnology, Santa Cruz, CA, USA) and anti-CD-11b (1:200; Abcam, Cambridge, UK). After 12 h, the membrane was washed with Tris-buffered saline with 0.1% Tween 20. Finally, the samples were incubated with horseradish peroxidase-conjugated anti-rabbit (1:5000; Leadgene Biomedical, Tainan, Taiwan) for four hours at 30 degrees and identified using Enhanced Chemi-luminescence Western Blotting Kit (Advansta, Menlo Park, CA, USA). The intensity of blots was quantified using the ImageJ software.

### 2.7. Quantitative Real-Time PCR

On day 7 after osmotic pump installation, animals were sacrificed and perfused through the aorta with normal saline. The lower lumbar enlargement (L4-6) spinal cord dorsal horn was separated. The total RNA was isolated using RNAiso Plus (Takara Bio Inc., Shiga, Japan) by following the guidelines supplied by the manufacturer. The first-strand cDNA was produced using the PrimerScript RT Reagent kit with gDNA Eraser (Takara Bio Inc., Shiga, Japan). The sequences of the primers used for Nrf2, HO-1, and GAPDH were as listed in Table 1 below. The Bio-Rad iCycler qPCR equipment and TB Green Premix EX Taq II (Takara Bio Inc., Shiga, Japan) were utilized for qPCR. The cycling conditions for both primer pairs were as follows: 5 s at 95 °C and 30 s at 60 °C. The ratios of Nrf2 and HO-1 mRNA expression levels were compared with the GAPDH levels in control groups. Data are shown as mean ± standard deviation.

### 2.8. Immunofluorescence Studies

The dorsal quadrant of the lumbar spinal cord was carefully isolated and preserved in paraformaldehyde. First, the spinal cords were fixed with 4% formaldehyde for 8 h and then with 30% sucrose for 8 h. The spinal cords were paraffinized and sectioned (10 µM). The slides were incubated for 12 h at 4 °C with primary antibody, IBA1 antibody at 1:100. The slides were then stained with DAPI (Sigma-Aldrich, St. Louis, MO, USA) for 1 h and scanned with a Pannoramic 205 FLASH II slide scanner. Scanned images were procured by using case viewer software. Quantitative analyses were performed using the ImageJ software.

### 2.9. Statistical Analysis

The data are expressed as the mean ± SD. All graphical representations and statistical calculations were aided by GraphPad Prism version 6.01. Two-way ANOVA, Tukey’s multiple comparison test, and Student’s *t*-test were used to analyze the statistical significance.

## 3. Results

### 3.1. Body Weight, Blood Glucose Levels, and Paw Withdrawal Sensitivity in Diabetic Rats

**Figure 1:**
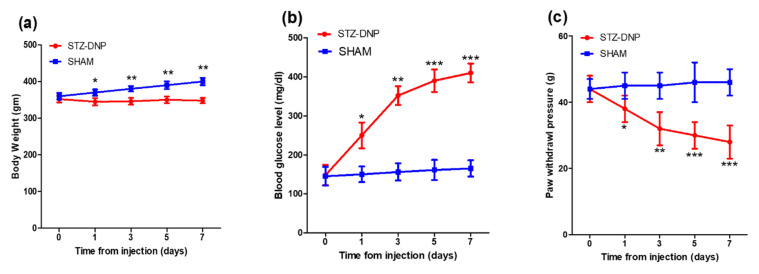
Changes in (**a**) body weight, (**b**) glycemic levels, and (**c**) paw withdrawal sensitivity in sham vs. DNP rats. Red curves indicate the mean ± SD of diabetic rats, and blue curves correspond to the mean ± SD of non-diabetic rats. Asterisk denotes a statistically significant difference when comparing sham rats vs. STZ DNP group. * *p* < 0.05; ** *p* < 0.01; *** *p* < 0.001 (*n* = 6 animals per group).

The blood glucose (BG) level and body weight (BW) were measured in both the diabetic group and non-diabetic group for 7 days. Body weight increase was significantly impaired in the diabetic group of rats compared with the non-diabetic group (day 7: 348 ± 18 vs. 400 ± 25 g; Figure 1a). Furthermore, the non-diabetic group maintained normal blood glucose levels, in comparison to high blood glucose levels in STZ-injected rats (day 7: 165 ± 21 vs. 410 ± 24 mg/dL; Figure 1b). The mean blood glucose level in all the rats that received an STZ injection met the norms for the diabetic category (i.e., ≥250 mg/dL). Furthermore, diabetic group rats displayed a significant decrease in paw withdrawal threshold 7 days after STZ injection, compared with the non-diabetic group, indicating the successful induction of diabetes (day 7: 28 ± 5 vs. 46 ± 4 g; Figure 1c).

### 3.2. The Antiallodynic and Anti-Hyperalgesic Effects of MOR in Diabetic and Non-Diabetic Rats

**Figure 2:**
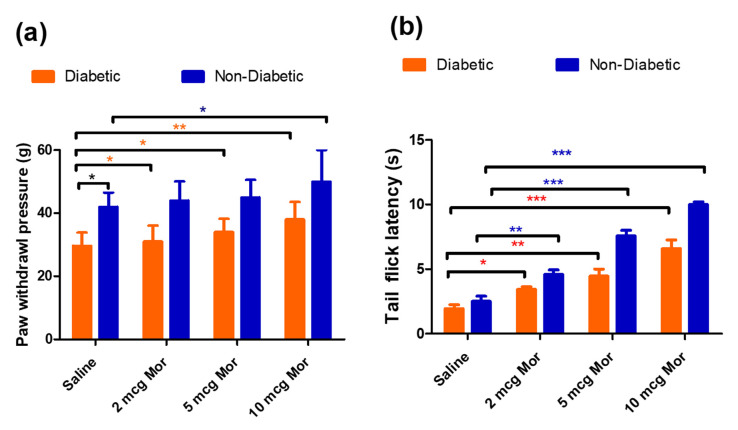
A single intrathecal injection of MOR was given in diabetic rats and non-diabetic rats at different doses. In diabetic rats, the drug was administered after 7 days of STZ injection. (**a**) Paw withdrawal response to mechanical allodynia in diabetic rats and non-diabetic rats, (**b**) tail-flick latency in response to hot water immersion test in diabetic rats and non-diabetic rats. Asterisk denotes a statistically significant difference when comparing saline rats vs. diabetic rats, saline diabetic rats vs. diabetic 2 mg MOR, saline diabetic rats vs. diabetic 5 mg MOR, and saline diabetic rats vs. diabetic 10 mg g MOR and a statistically significant difference when comparing saline non-diabetic rats vs. non-diabetic 2 mg MOR, saline non-diabetic rats vs. non-diabetic 5 mg MOR, and saline non-diabetic rats vs. non-diabetic 10 mg MOR. * *p* < 0.05; ** *p* < 0.01; *** *p* < 0.001 (*n* = 6 animals per group).

The antinociceptive effects of MOR at different doses were tested in diabetic rats (7 days after STZ injection) and non-diabetic rats through a single intrathecal injection of MOR or saline at different doses (2, 5, and 10 μg, i.t) followed by the measurement of tail-flick latency and mechanical paw withdrawal threshold after 60 min of MOR injection. At the baseline, even before giving any MOR injection, the diabetic rats displayed a significant reduction in paw withdrawal threshold (29 g ± 4.2 g vs. 42 ± 4.5 g) when compared to saline rats. However, no significant difference was noticed in tail-flick tests (1.9 ± 0.3 s vs. 2.5 ± 0.4 s). When compared to diabetic rats, the non-diabetic group showed an enhanced response to MOR at all the tested doses (2, 5, and 10 μg, i.t) in both paw withdrawal threshold (Figure 2a) and tail-flick latency tests (Figure 2b). This effect might be attributed to the decrease in the opioid receptors in the spinal cord of diabetic animals, which in turn affects the antinociceptive effects [37].

### 3.3. Effects of Spinal Infusion of MOR, TEN, and Their Combination on Tactile Allodynia and MAT

**Figure 3:**
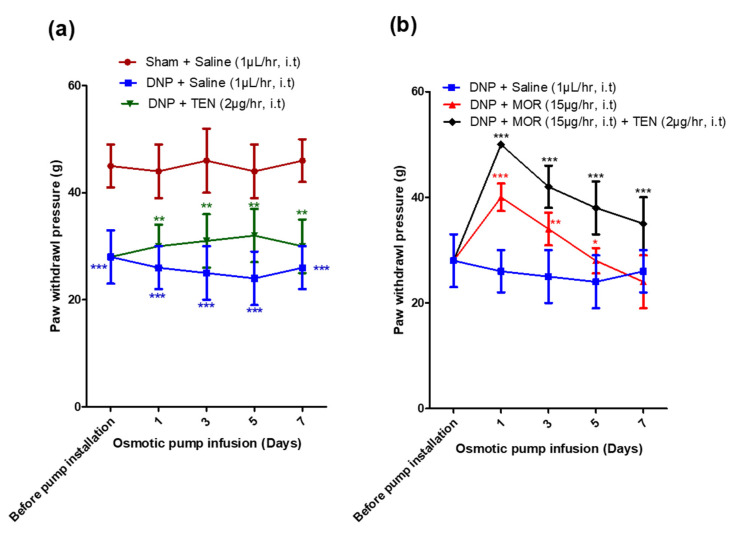
Changes in mechanical paw withdrawal thresholds of rats after installation of osmotic pump after establishment of DNP in (**a**) sham + saline rats, DNP + saline rats, and rats infused with DNP + TEN. (**b**) DNP + saline, DNP + MOR, and DNP + MOR + TEN. Before pump installation readings denote day 7 readings after STZ injection (*n* = 6). The asterisk in (**a**) denotes a statistically significant difference when comparing sham vs. DNP + saline and DNP + saline vs. DNP + TEN. Asterisk in (**b**) denotes a statistically significant difference when comparing DNP + saline vs. DNP + MOR and DNP + MOR vs. DNP + TEN + MOR. * *p* < 0.05; ** *p* < 0.01; *** *p* < 0.001 (*n* = 6 animals per group).

The sham group of rats had less paw withdrawal sensitivity than the DNP groups (45 ± 4 vs. 28 ± 5 g; Figure 3a, brown, green and blue curves) before osmotic pump infusion, suggesting the successful establishment of DNP in STZ-injected rats. Intrathecal infusion of TEN alone at 2 µg/h displayed mild analgesic effects compared to the DNP saline group (Figure 3a, green curve). Intrathecal infusion of MOR alone at a dose of 15 µg/h resulted in a significant reduction in tactile allodynia in DNP rats compared to the saline-infused DNP group on day 1 of infusion (40 ± 3 vs. 26 ± 4 g; Figure 3b, red curve). However, there was a significant decrease in the paw withdrawal threshold in the MOR-infused group on day 7 of infusion due to the opioid-induced tolerance, with a paw withdrawal threshold of 24 ± 5 g which is not significantly different from that of the DNP saline-infused group (Figure 3b, red and blue curves). When MOR was infused in combination with 2 µg/h of TEN, it enhanced the antinociceptive effects of MOR (50 ± 0 g vs. 40 ± 3 g; Figure 3b, black curve). MOR co-infusion with TEN significantly increased the paw withdrawal threshold when compared to MOR infusion alone on day 7 (35 ± 5 g vs. 24 ± 5 g), signifying a reduction in MAT (Figure 3b, black curve).

### 3.4. Effects of Spinal Infusion of MOR, TEN, and Their Combination on Blood Glucose Levels

**Figure 4:**
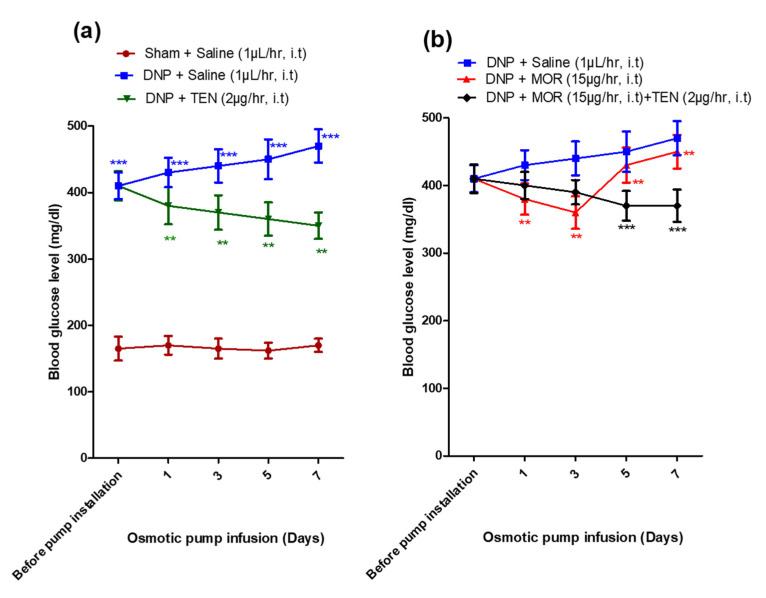
Changes in blood glucose level of rats after installation of the osmotic pump in (**a**) sham + saline, DNP + saline, and DNP + TEN and (**b**) DNP + saline, DNP + MOR, and DNP + MOR + TEN. The asterisk in (**a**) denotes a statistically significant difference when comparing sham vs. DNP + saline and DNP + saline vs. DNP + TEN. Asterisk in Figure (**b**) denotes a statistically significant difference when comparing DNP + saline vs. DNP + MOR and DNP + MOR vs. DNP + TEN + MOR. ** *p* < 0.01; *** *p* < 0.001 (*n* = 6 animals per group).

The sham group of rats displayed lower blood glucose levels than the DNP groups (165 ± 18 vs. 410 ± 22 mg/dL, Figure 4a, brown, blue, and green curves) before osmotic pump infusion, suggesting the successful establishment of DNP in STZ-injected rats. Intrathecal infusion of TEN alone at 2 µg/h displayed mild glucose-lowering effects compared to DNP saline (Figure 4a, green curve). MOR infusion slightly reduced the blood glucose levels during the initial 3 days, compared to DNP saline. However, the anti-hyperglycemic effect gradually diminished on day 7 (day 3, 360 mg/dL vs. day 7, 450 mg/dL; Figure 4b, red curve). When compared to MOR infusion alone, the co-infusion with TEN significantly reduced the blood glucose levels (day 7, 450 mg/dL vs. 370 mg/dL, Figure 4b, black curve).

### 3.5. Effects of Spinal Infusion of MOR, TEN, and Their Combination on Body Weight in Diabetic Rats

**Figure 5:**
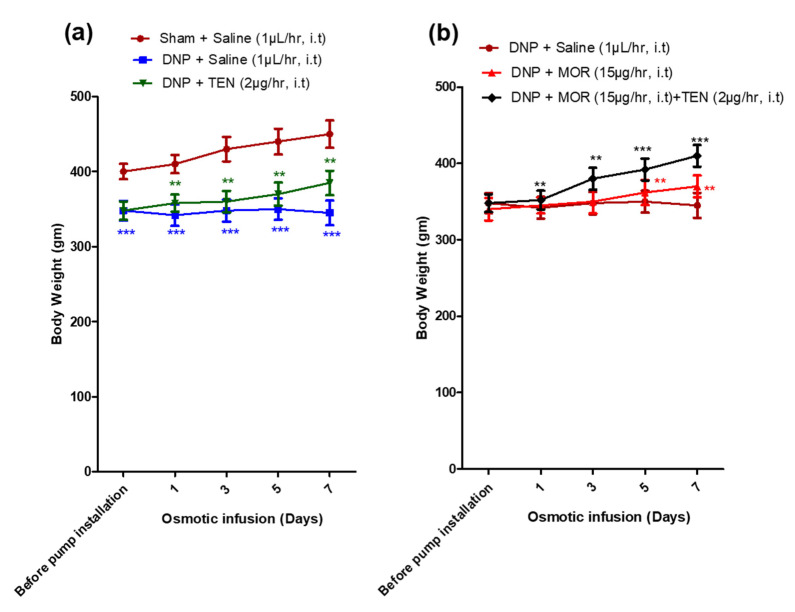
Changes in body weight level of rats after installation of the osmotic pump in (**a**) sham + saline, DNP + saline, and DNP + TEN and (**b**) DNP + saline, DNP + MOR, and DNP + MOR + TEN. The asterisk in (**a**) denotes a statistically significant difference when comparing sham + saline vs. DNP + saline and DNP + saline vs. DNP + TEN. Asterisk in Figure (**b**) denotes a statistically significant difference when comparing DNP + saline vs. DNP + MOR and DNP + MOR vs. DNP + TEN + MOR. ** *p* < 0.01; *** *p* < 0.001 (*n* = 6 animals per group).

The sham group of rats displayed significantly lower body weight than the DNP groups (348 ± 30 g vs. 435 ± 35 g; Figure 5a, brown, blue, and green curves) before osmotic pump infusion, suggesting the successful establishment of DNP in STZ-injected rats. Intrathecal infusion of TEN alone at 2 µg/h displayed mild body weight restoration compared to the DNP saline group (Figure 5a, green curve). MOR infusion slightly restored the body weight levels on day 5 and day 7 of infusion (Figure 5b, red curve). When compared to MOR infusion alone, the co-infusion with TEN significantly restored the body weight levels (Figure 5b, black curve).

### 3.6. Teneligliptin Suppressed MOR-Induced Nrf-2 Inflammasome Activation and HO-1 Activation in the Dorsal Horn of Diabetic Rats

**Figure 6:**
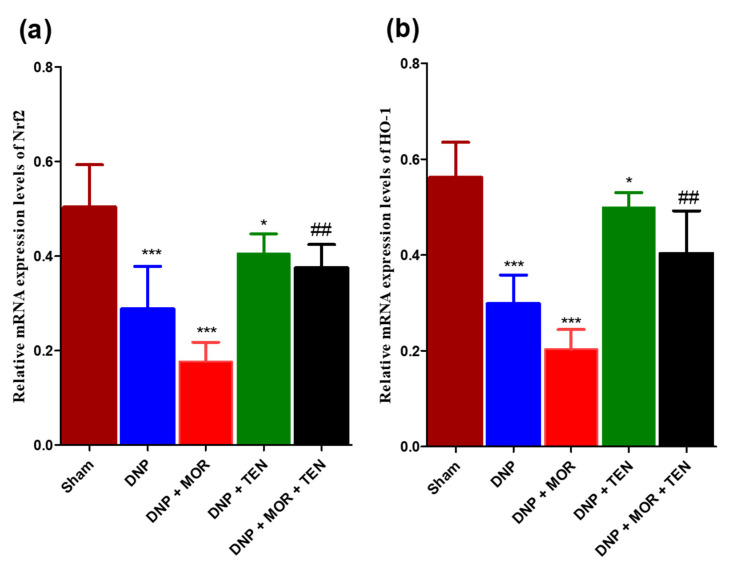
Relative mRNA expression levels of (**a**) Nrf2 and (**b**) HO-1 were determined by Q-PCR. The asterisk denotes a statistically significant difference when comparing sham + saline vs. DNP + saline and DNP + saline vs. DNP + TEN and DNP + saline vs. DNP + MOR. # denotes a statistically significant difference when comparing DNP + MOR vs. DNP + MOR + TEN. * *p* < 0.05; ## *p* < 0.01; *** *p* < 0.001 (*n* = 6 animals per group).

The mRNA protein levels of Nrf2 and HO-1 were measured in the dorsal horn of sham and diabetic rats infused with vehicle, MOR, TEN, or MOR + TEN for 7 days and are shown in Figure 6. Our results demonstrated that the expression of both Nrf2 and HO-1 was reduced in the dorsal horn of DNP rats compared to the sham group (Figure 6a,b, brown and blue bars). TEN alone was efficient in restoring the Nrf-2 and HO-1 levels in DNP rats when compared to the DNP + saline group (Figure 6a,b, green bars). MOR infusion reduced the expression of Nrf2 and HO-1 significantly when compared to the DNP + saline group (Figure 6a,b, red bars). Co-infusion of TEN with MOR effectively restored the Nrf-2 and HO levels when compared to MOR infusion alone (Figure 6a,b, black bars).

### 3.7. Teneligliptin Suppressed MOR-Induced CD-11b Expression in the Dorsal Horn of Diabetic Rats

**Figure 7:**
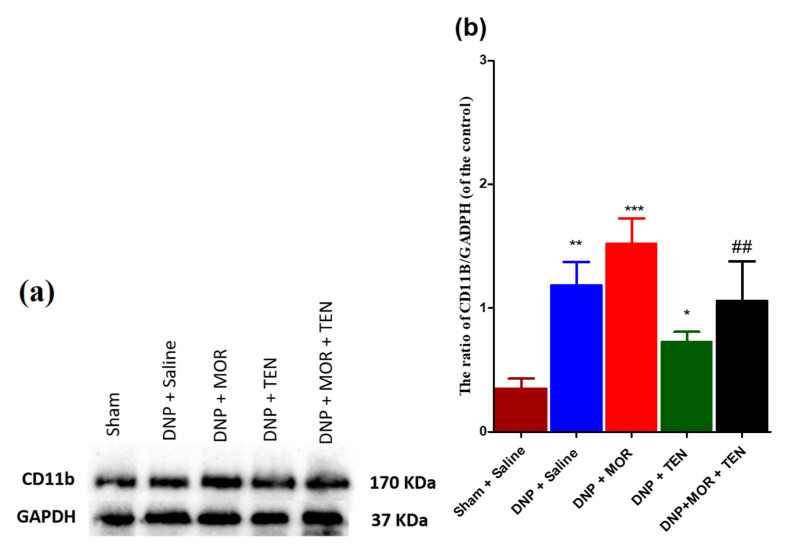
The expression and activation of CD-11b. (**a**) Western blot and (**b**) the quantitative analysis of CD11b in sham and DNP rat models receiving i.t infusion with saline (1 µL/h), MOR (15 μg/h), TEN (2 μg/h), or MOR (15 μg/h) + TEN (2 μg/h) for 7 days. The asterisk denotes a statistically significant difference when comparing sham + saline vs. DNP + saline, DNP + saline vs. DNP + TEN, and DNP + saline vs. DNP + MOR. # denotes a statistically significant difference when comparing DNP + MOR vs. DNP + MOR + TEN. * *p* < 0.05; **/## *p* < 0.01; *** *p* < 0.001 (*n* = 6 animals per group).

Western blot assays have established that DNP increased the activation of microglial cells when compared to saline rats (Figure 7a, second lane, and Figure 7b, blue bar). Intrathecal infusion of MOR significantly increased the microglial cell activation, manifested as robust upregulation of microglial marker CD-11b in the dorsal horn when compared to DNP + saline rats (Figure 7a, third lane, and Figure 7b, red bar). In DNP rats, TEN infusion alone significantly decreased microglial cell activation when compared to DNP + saline rats (Figure 7a, fourth lane, and Figure 7b, green bar). MOR in combination with TEN significantly reduced the glial cell activation when compared to infusion of MOR alone (Figure 7a, fifth lane, and Figure 7b, black bar). These results indicate that TEN significantly attenuates the microglial activation either alone or in combination with MOR.

### 3.8. TEN Inhibits Microglial Activation in the Dorsal Horn of DNP and MOR-Infused DNP Rats

**Figure 8:**
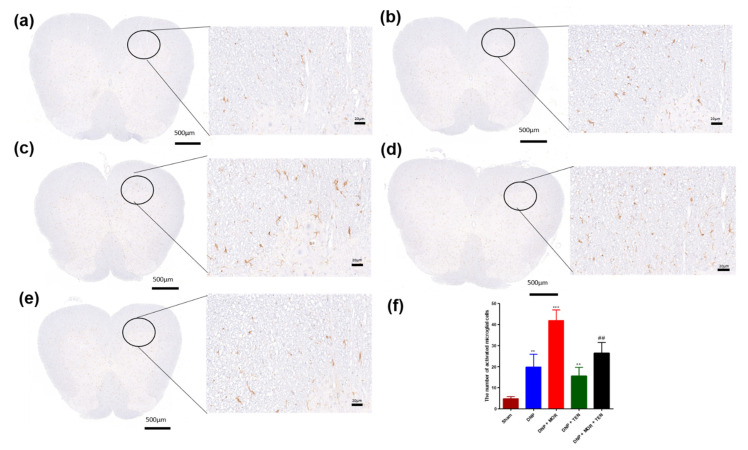
Activated microglial cells using IBA-1 antibody in (**a**) sham + saline-, (**b**) DNP + saline-, (**c**) DNP + MOR-, (**d**) DNP + TEN-, and (**e**) DNP + MOR + TEN-infused rats. (**f**) The quantitative analysis of activated microglial cells. There was a significant increase in activated microglia after MOR infusion which was ameliorated by TEN co-infusion. All spinal cord samples were collected on day 7 after the osmotic pump infusion. The asterisk denotes a statistically significant difference when comparing sham vs. DNP + saline, DNP + saline vs. DNP + TEN, and DNP + saline vs. DNP + MOR. # denotes a statistically significant difference when comparing DNP + MOR vs. DNP + MOR + TEN. **/## *p* < 0.01; *** *p* < 0.001 (*n* = 6 animals per group). Scale bar = 20 µm.

To examine the inhibitory effect of TEN on DNP and MOR-induced microglial cell activation in the dorsal horn, immunohistochemistry was performed with microglial cell-specific antibody IBA-1, 7 days after infusion with TEN, MOR, or MOR + TEN in DNP rats. Microglial cell number was counted in the sections of the spinal cord dorsal horn. The morphology of IBA-1-positive cells in sham rats displayed small dot-like structures, indicating a resting microglial cell state (Figure 8a,f, brown bar). After DNP establishment, IBA-1-positive cells displayed a slightly increased cell body hypertrophy when compared to sham rats (Figure 8b,f, blue bar). TEN infusion alone was successful in the alleviation of microglial cell activity in DNP rats when compared to DNP + saline (Figure 8d,f, green bar). MOR infusion greatly aggravated the activation of microglia with ameboid morphology indicating hyperactive microglial cells compared to DNP saline (Figure 8c,f red bar). TEN in combination with MOR significantly attenuated MOR-induced microglial cell activation when compared to MOR infusion alone (Figure 8e,f, black bar). These results indicate that microglia were activated in the spinal dorsal horn of DNP rats, MOR infusion further aggravated the activation of microglial cells, and TEN co-infusion with MOR significantly attenuated microglial cell activation by reversing the pathological changes

### 3.9. TEN Inhibits Mononucleate Cell Infiltration in the Dorsal Horn of DNP and MOR-Infused DNP Rats

**Figure 9:**
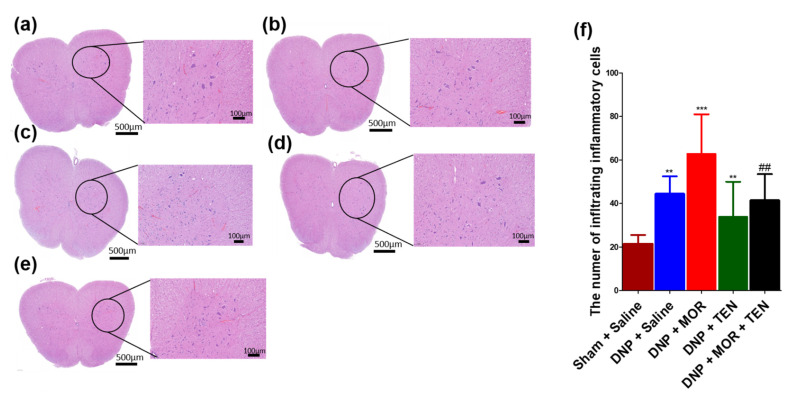
H&E staining showing mononucleate cell infiltration, a marker of inflammatory response in (**a**) sham + saline-, (**b**) DNP + saline-, (**c**) DNP + MOR-, (**d**) DNP + TEN-, and (**e**) DNP + MOR + TEN-infused rats. (**f**) The quantitative analysis of infiltrates. MOR infusion aggravated mononuclear cell infiltration. TEN infusion in combination with MOR alleviated the inflammatory response. All spinal cord samples were collected on day 7 after the osmotic pump infusion. * denotes a statistically significant difference when comparing sham + saline vs. DNP + saline and DNP + saline vs. DNP + TEN. # denotes a statistically significant difference when comparing DNP + MOR vs. DNP + MOR + TEN and DNP + saline vs. DNP + MOR. **/## *p* < 0.01; *** *p* < 0.001 (*n* = 6 animals per group). Scale bar = 100 µm.

We next examined the pathological aspects of DNP and MAT in the sham, DNP, or DNP + TEN treatment and DNP + MOR or DNP + TEN + MOR treatment, focusing on mononucleate immune cell infiltration in the L4 sections of the dorsal horn. Upon H&E staining, the L4 section of the sham group showed little or no immune cell infiltration (Figure 9a,f, brown bar), whereas the DNP showed a slight increase in immune cell infiltration (Figure 9b,f, blue bar). The spinal cords of TEN-infused rats showed less immune cell infiltration compared to DNP + saline-infused rats (Figure 9d,f, green bar). MOR-infused rats showed immense immune cell infiltration (Figure 9c,f, red bar) compared to DNP + saline rats. Co-infusion of TEN in combination with MOR significantly reduced MOR-induced immune cell infiltration (Figure 9e,f, black bar).

## 4. Discussion

The prescription of opioid analgesics for the management of NP has been a subject of current debate in preclinical and clinical studies. Though opioids, such as MOR, remain the “gold standard” for acute pain relief, they are known to have limited efficacy in treating chronic pain conditions such as DNP, a prevalent complication in patients with DM. It is well documented that the antinociceptive action of MOR is reduced in both preclinical and clinical models of DNP due to a reduction in the number of opioid receptors or the impairment of µ-opioid receptor–G protein coupling [37,38,39,40]. Likewise, the density of opioid receptors is significantly decreased in the spinal dorsal horn of animals with DNP [41]. Fluctuations in the concentration of either brain or blood glucose levels are known to modulate opioid antinociception and basal nociceptive processing in animal models [42,43]. Some studies also suggest that DM can directly affect beta-endorphin synthesis and beta-endorphin concentration in the CNS, which can alter MOR response in DNP rats [44]. Additionally, a significant upregulation of L-type Ca^2+^ channels in the CNS of DNP rats is known to attenuate MOR’s antinociception [45]. Our results are in agreement with the previous reports, suggesting the decreased antinociceptive action of MOR in DNP rats [46].

The analgesic potential of DPP-4i TEN has been previously investigated by our group in a partial sciatic nerve transection model of neuropathic pain [34]. In clinical practice, most DPP4-is are used as antidiabetic drugs, but recent studies have confirmed the role of DPP4-is in the CNS. DPP-4is such as omarigliptin are effective in penetrating the BBB due to their low molecular weight and lipophilic properties, with potent properties for repurposing as an antiparkinson drug [47]. Though DPP-4is such as linagliptin do not possess the ability to cross the BBB, they are still proven to possess neuroprotective effects, especially in Alzheimer’s disease, by increasing the bioactivity of GLP-1, which is effective in penetrating the BBB [48]. Similarly, other DPP4-i including saxagliptin, vildagliptin, and sitagliptin were effective in increasing GLP-1 expression in the hippocampus of various animal models of Alzheimer’s disease, although these drugs are not efficient in crossing the BBB [49,50,51]. Recently, some research evidence suggested the involvement of GLP-1 receptors in MOR dependence and withdrawal symptoms [52]. Treatment with linagliptin, a selective DPP4-i, inhibited both short-term and long-term MOR withdrawal symptoms in mice [52]. Another study reported the efficacy of linagliptin in reducing MOR’s rewarding effect in rats [53]. However, there are no preclinical or clinical studies that evaluated the efficacy of DPP4-is in combination with MOR.

The present study was designed to examine the effects of TEN co-infusion on the analgesic efficacy of MOR and the development of MAT in DNP rats by measuring paw withdrawal threshold in response to mechanical stimulus and tail-flick tests. TEN co-infusion at a dose of 2 µg/h with MOR 2 µg/h significantly enhanced the analgesic activity of MOR (100% vs. 80% on day 1 of infusion) in DNP rats. In comparison, infusion of TEN alone showed mild analgesic activity compared to saline-infused DNP rats. Additionally, we have demonstrated that intrathecal co-infusion of TEN significantly decreased MAT. To delineate the underlying mechanisms of enhanced analgesic action of MOR and MAT alleviation, we have measured the mRNA levels of Nrf-2 and HO-1, as several DPP-4is are known to activate the expression of various neuroprotective genes that code for antioxidant and anti-inflammatory activities, in particular, Nrf-2 and HO-1 [54,55,56,57,58]. Our results suggested that TEN significantly restores the mRNA levels of neuroprotective proteins Nrf2 and HO-1 in the spinal dorsal horn of DNP rats when co-infused with MOR. Previous studies indicate that the activation of Nrf2 and HO-1 can enhance MOR’s analgesic activity through various mechanisms. For example, sulforaphane is known to activate Nrf2 and enhance the antiallodynic and anti-hyperalgesic activity of MOR. This enhancement of analgesic action is ascribed to the upregulation of μ-opioid receptors and HO-1 in the spinal cord [59]. Validating these findings, an HO-1 inducer, cobalt protoporphyrin IX, decreased DNP and enhanced the antinociceptive action of MOR in DNP mice [60]. Additionally, DPP-4is are known to have various pleiotropic effects, such as anti-apoptotic signaling, inhibition of toxic protein phosphorylation, neuroprotection, and enhancing synaptic plasticity, which are also key players in reducing MAT [61,62,63,64].

Non-neuronal cells, particularly microglia, play an important role in both DNP and MAT [65]. Both DNP and chronic MOR exposure are known to strongly activate microglial cells [66,67]. Current literature suggests that microglial cell activation is associated with cytokine release in the central nervous system [68]. Both chronic and acute exposure to MOR can activate microglial cells, which in turn leads to the increased expression of PICs, such as IL-1β, IL-6, and TNF-α [68]. The release of PICs can lead to MAT. Microglial cell inhibition in the dorsal horn had been shown to attenuate MAT and DNP in various animal models [8,69,70].

In the current study, we found that DNP can cause microglial cell activation, and MOR infusion can further enhance microglial cell activation. Co-infusion of TEN inhibited MOR-induced microglial cell activation. These data suggest that TEN co-infusion may be effective in reducing microglial activation in both DNP and MAT.

The activation of microglial cells and the release of PICs can lead to the infiltration of immune cells like macrophages and lymphocytes into the site of spinal cord injury as a mechanism for neuroprotection [71]. The infiltration or migration of immune cells to the site of injury is considered as a hallmark of inflammation as a part of innate immune response [71]. Both DNP and MAT are known to cause neuroinflammation and immune cell infiltration in the dorsal horn [72,73,74,75]. Our results indicated that TEN co-infusion can significantly alleviate MOR-induced glial cell activation and immune cell infiltration in the spinal cord dorsal horn. This study demonstrated important results in terms of the effects of TEN on MOR’s antinociceptive action and MAT in DNP rats. Further studies are needed to fully delineate the underlying mechanisms of the antinociceptive action of TEN on MAT in animals with DNP.

## 5. Conclusions

In summary, our study results indicate that co-infusion of TEN increased the antinociceptive efficacy of MOR and attenuated MAT. Additionally, TEN co-infusion increased the expression of mRNA levels of Nrf-2 and HO-1 and decreased glial cell activation and immune cell infiltration in the spinal dorsal horn of DNP rats. Our work lays a foundation for prospective clinical translation of TEN for the effective management of MAT in patients with chronic pain and DNP. Further investigation is necessary to confirm the possible role of DPP4i and its effect on MOR’s antinociceptive action and MAT in DNP rats.

## Figures and Tables

**Table 1:** Sequences of the primers used for Nrf2, HO-1, and GAPDH.

Gene	Sequence of Primers
Nrf-2	Forward: 5′-TTGGCAGAGACATTCCCATTTGTA-3′ Reverse: 5′-GAGCTATCGAGTGACTGAGCCTGA-3′
HO-1	Forward: 5′-AGGTGCACATCCGTGCAGAG-3′ Reverse: 5′-CTTCCAGGGCCGTATAGATATGGTA-3′
GAPDH	Forward: 5′-GGCACAGTCAAGGCTGAGAATG-3′ Reverse: 5′-ATGGTGGTGAAGACGCCAGTA-3′

## Data Availability

The data presented in this study are available within the article.
